# Hesperetin Nanoparticle Powder as a Potential Antioxidant Nutraceutical Ingredient: Fabrication, Characterization, and Comparative Dissolution in Vegetarian and Non-Vegetarian Capsules

**DOI:** 10.3390/pharmaceutics17121558

**Published:** 2025-12-03

**Authors:** Tzu-Hui Wu, Yun-Yi Lan, Huai-En Hsu, Pamela Berilyn So, Yuan-Yu Chen, Feng-Lin Yen

**Affiliations:** 1 Department of Pharmacy and Master Program, College of Pharmacy and Health Care, Tajen University, Pingtung 90741, Taiwan; u93070@tajen.edu.tw (T.-H.W.); jerry1233793@gmail.com (Y.-Y.L.); a5795026@gmail.com (H.-E.H.);; 2 Department of Fragrance and Cosmetic Science, College of Pharmacy, Kaohsiung Medical University, Kaohsiung 807378, Taiwan; pam@kmu.edu.tw; 3 Department of Medical Research, Kaohsiung Medical University Hospital, Kaohsiung 807378, Taiwan; 4 Institute of Biomedical Sciences, National Sun Yat-Sen University, Kaohsiung 80424, Taiwan; 5 Drug Development and Value Creation Research Center, Kaohsiung Medical University, Kaohsiung 80756, Taiwan; 6 College of Professional Studies, National Pingtung University of Science and Technology, Pingtung 912301, Taiwan

**Keywords:** hesperetin, nanoparticles, water solubility, gelatin (non-vegetarian) and HPMC (vegetarian) capsules, dissolution

## Abstract

**Background/Objectives:** Hesperetin (HSP) is a bioactive flavonoid known for its strong antioxidant and anti-inflammatory properties. However, its low water solubility (1.36 ± 0.30 μg/mL) and poor oral bioavailability (~20%) greatly hinder its potential in nutraceutical applications. **Methods:** Using the solvent dispersion method, nanoparticles composed of HSP, hydroxypropyl-β-cyclodextrin (HPBCD), and polyvinylpyrrolidone K30 (PVPK30) were prepared and collectively termed HHPNP. Characterization involved particle size measurement, FTIR, XRD, SEM, and TEM. Antioxidant activity was evaluated using DPPH and ABTS^+^ radical scavenging assays. In vitro dissolution testing was performed at pH 1.2 and pH 6.8 to compare HHPNP with physical mixtures, and release behavior was assessed using both gelatin (non-vegetarian) and HPMC (vegetarian) capsules. **Results:** The optimal formulation (1:15:12) produced uniformly distributed spherical nanoparticles with a mean size of 14.87 ± 0.49 nm and achieved an 827-fold increase in water solubility compared with raw HSP. FTIR analysis indicated hydrogen bond formation, and XRD confirmed a complete transition from a crystalline to an amorphous state. In aqueous environments, HHPNP demonstrated markedly improved antioxidant activity, with DPPH and ABTS^+^ radical scavenging comparable to HSP solutions prepared in methanol. In vitro dissolution testing revealed rapid release at both pH 1.2 (>65% in 10 min) and at pH 6.8 (70% in 5 min). In contrast, physical mixtures only released 10–30% over two hours. T50% values at pH 1.2 were 17.8 min (gelatin) and 16.8 min (HPMC). At pH 6.8, T50% values were 17.6 min (gelatin) and 7.5 min (HPMC). Both capsule types matched the HHPNP in release at 120 min, and these comparable profiles indicate the formulation’s stability and adaptability across capsule variants. **Conclusions:** This nanoparticle-based delivery system, leveraging molecular inclusion and amorphization, significantly enhanced the solubility, bioactivity, and release efficiency of HSP, offering a potent platform for oral flavonoid-based dietary supplements.

## 1. Introduction

Nutraceuticals, such as functional foods and dietary supplements, primarily derived from natural sources, are mainly used to improve human health and prevent diseases. Due to population aging and the increasing prevalence of diseases, the global nutraceuticals market reached a value of USD 472.7 billion in 2022, with a compound annual growth rate of 8.9%. With this continuous growth, it is expected to exceed USD 1 trillion by 2032 [1]. Flavonoids are naturally occurring compounds featuring a flavanone core composed of two aromatic rings, connected by a dihydro-2H-pyran-3-one ring. Flavonoids are widely present in fruits, vegetables, tea, and legumes, and are recognized as important dietary nutraceuticals. Over 4000 flavonoids have been identified, exhibiting various biological activities such as antioxidant, anticancer, anti-inflammatory, and immunomodulatory effects [2].

**Figure 1:**
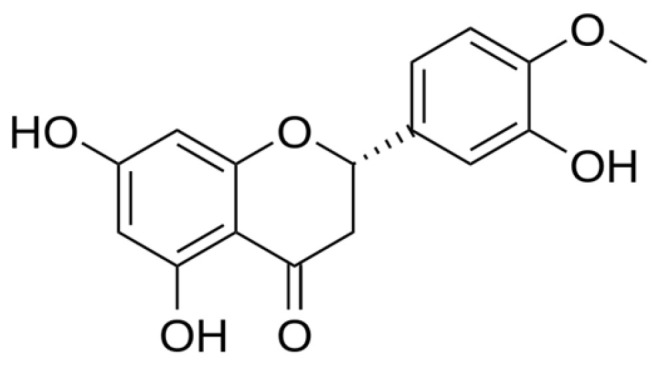
Chemical structure of hesperetin.

Hesperetin (3′,5,7-trihydroxy-4′-methoxyflavanone, HSP, Figure 1) is a flavonoid with notable antioxidant, anti-inflammatory, antitumor, anti-aging, and neuroprotective properties [3]. It is Generally Recognized as Safe (GRAS) by the U.S. FDA. The global HSP market is estimated to be at USD 240 million in 2024 and projected to reach USD 500 million by 2033 [4]. In addition, employing Lipinski’s rule of five, and in silico simulations for the preliminary screening of HSP, demonstrated favorable gastrointestinal absorption, indicating considerable potential for further development. However, it should be noted that HSP exhibits extremely low water solubility (0.45 ± 0.01 × 10^−5^ mol/L equal to 1.36 ± 0.30 μg/mL), which significantly affects its absorption, gastrointestinal stability, and overall bioavailability, which is reported to be approximately 20% [5,6]. As a hydrophobic flavonoid, HSP’s poor solubility and limited oral absorption substantially restrict its pharmacological efficacy. [7].

Over the past few decades, nanoparticle delivery systems, such as polymer nanoparticles, micelles, liposomes, solid lipid nanocarriers, nanostructured lipid carriers, nanoemulsions, and inclusion complexes, have been widely used to improve the water solubility and oral bioavailability of highly lipophilic nutraceuticals, such as curcumin [8], lycopene [9], and quercetin [10]. In addition, existing nutraceuticals in the market are mostly filled in capsules to improve the stability of nutraceuticals in the gastrointestinal tract, as well as to reduce first-pass metabolism in the intestine and liver, further improving oral bioavailability and bioactivity [11,12].

This study proposes that HSP loaded hydroxypropyl-β-cyclodextrin (HPBCD) and polyvinylpyrrolidone K30 (PVPK30) nanoparticle powder (HHPNP) can enhance HSP delivery, by increasing water solubility, improving encapsulation efficiency, increasing in vitro release rate, maintaining antioxidant capacity, and ensuring good release stability across different capsule types. This study utilizes a laser particle size analyzer, scanning electron microscope (SEM), powder X-ray diffraction (XRD), Fourier-transform infrared spectroscopy (FTIR), high-performance liquid chromatography (HPLC) to analyze the physicochemical properties of the HHPNP formulation, and an in vitro antioxidant experiment to confirm its antioxidant activity. Additionally, the HSP release rates of the HHPNP in non-vegetarian (gelatin) and vegetarian (HPMC) capsules remain unexplored. This study compares in vitro dissolution profiles of HSP, HHPNP, and HHPNP filled in gelatin and HPMC capsules to confirm HHPNP’s efficacy as an advanced flavonoid-based delivery nanocarrier, highlighting its potential for use in functional nutritional supplements.

## 2. Materials and Methods

### 2.1. Materials

Hesperetin (purity ≧ 98%) was obtained from Baichuan Kanze Co., Ltd. (Shaanxi, China). 2-hydroxypropyl-β-cyclodextrin (HPBCD) with an average molar substitution degree of 4.75 per anhydroglucose unit (average molecular weight 1410 g/mol) was purchased from Zibo Qianhui Co., Ltd. (Shandong, China). Polyvinyl-N-pyrrolidone K30 (PVPK30) with average molecular weight 40,000 g/mol), 2,2-diphenyl-1-picrylhydrazyl (DPPH) (≥99%) and 2,2′-azino-bis-3-ethylbenzaothazoline-6-sulfonic acid (ABTS) (≥98%) were obtained from Sigma-Aldrich Co., Ltd. (St. Louis, MO, USA). All chemicals used were of high-performance liquid chromatography (HPLC) grade.

### 2.2. Preparation of Hesperetin Loaded HPBCD and PVPK30 Nanoparticle Powder (HHPNP)

HHPNP nanoparticles were prepared using the solvent dispersion method, investigating the effects of composition by examining the weight ratios (*w*/*w*/*w*) of HSP, HPBCD, and PVPK30D at 1:15:5, 1:15:12, and 1:15:15 (Table 1). To formulate the solution, 50 mg of raw HSP was initially dissolved in 10 mL of ethanol. Subsequently, HPBCD were incorporated into the mixture under continuous stirring for 1 h. Following this step, PVPK30 were added to the HSP/HPBCD solution, ensuring steady agitation for an additional hour to achieve thorough integration. The ethanol present in the solution was carefully evaporated at 50 °C with the aid of a rotary vacuum system, resulting in the formation of a dry, white-yellow nanoparticle powder, denoted as HHPNP. The obtained nanoparticles were then stored in a moisture-resistant container to preserve their integrity for future applications. For comparison, physical mixtures of HSP, HPBCD, and PVPK30 were also prepared in ratios of 1:15:5, 1:15:12, and 1:15:15, then mixed for one minute.

### 2.3. Establishment of a Calibration Curve for Hesperetin

HSP solutions (0.5–100 μg/mL) in methanol were used to generate a calibration curve via HPLC (L-5000, Hitachi, Tokyo, Japan) equipped with a L-5160 pump, a L-5260 autosampler, a L-5310 column oven, a L-5430 (UV–vis) detector, and a reverse-phase C18 column (Inertsil ODS-3, 5 μm, 4.6 × 250 mm). The mobile phase was methanol and 0.1% phosphoric acid (70:30, *v*/*v*). Each 20 μL sample was analyzed at 280 nm under a 1 mL/min flow rate. HSP exhibited a retention time of 5.327 min, with linear regression calibration (r^2^ > 0.999) employed for solubility, encapsulation efficiency, and dissolution studies.

### 2.4. Determination of Yield and Encapsulation Efficiency of HHPNP

The yield and encapsulation efficiency of HHPNP serve as pivotal metrics for assessing the successful manufacturing process. To evaluate the yield of HHPNP, various formulations with differing ratios were dissolved in methanol, ensuring thorough solvation. Each sample underwent vigorous mixing for 10 min prior to measurement of HSP concentration using the previously described HPLC method. This analysis provided precise calculations of HSP yield, enabling an accurate assessment of manufacturing outcomes. The yield is calculated using the formula given in Equation (1).

Yield (%) = CRNP/CR × 100%(1)

where CR: Theoretical amount of HSP, CRNP: HSP concentration in HHPNP after HPLC analysis.

To evaluate the encapsulation efficiency of HHPNP, each sample was dissolved in distilled water and subjected to mixing using a shaker for 10 min. Subsequently, 200 μL of the prepared samples were transferred into a centrifugal filter (Nanosep^®^ Centrifugal Devices with Omega™ Membrane, 10,000 molecular weight; Pall Corporation, Port Washington, NY, USA) and centrifuged (Model 5430 R; Eppendorf, Hamburg, Germany) at a speed of 3500 rpm for 15 min. During this process, the encapsulated portion was retained in the upper chamber of the filter, whereas the unencapsulated HSP fraction of HHPNP passed through to the lower chamber. The concentration of HSP in each sample was subsequently analyzed using the previously described HPLC technique. Afterwards, Equation (2) was employed to calculate the encapsulation efficiency of HHPNP.

Encapsulation efficiency (%) = CR − unencapsulated HSP fraction of HHPNP/CR × 100%(2)

### 2.5. Water Solubility Analysis

Raw HSP (1 mg) and HHPNP (equivalent to 1 mg of HSP) were added in 1 mL of distilled water separately. Each sample underwent vigorous shaking using a mixer (Vortex-Genie 2, Scientific Industries, Bohemia, NY, USA) for 10 min, followed by filtration through a 0.45 μm syringe filter (13 mm Acrodisc^®^ syringe filters with GHP membrane; Pall Corporation, Port Washington, NY, USA). Afterwards, the samples were analyzed using the previously described HPLC method, and the solubility of HSP was determined.

### 2.6. Particle Size Analysis

The particle size and polydispersity index of the raw HSP and HHPNP formulations were analyzed using the Zetasizer Nano-ZS ZEN3600 (Malvern Instruments Ltd., Malvern, UK). Each sample containing 1 mg of HSP per mL of distilled water was diluted 10-fold and then determined at 25 °C with a detection angle of 90° in triplicate.

### 2.7. Scanning/Transmission Electron Microscopy (SEM/TEM)

The surface morphology of raw HSP, HHPNP, their physical blend, and excipients was examined using a scanning electron microscope (SEM; Hitachi S-4700, Tokyo, Japan). Prior to imaging, each sample was coated with a gold–palladium layer under an argon atmosphere using an ion sputter coater (Hitachi E-1045, Tokyo, Japan). The particle morphology of HHPNP was observed by diluting 1 mg of HSP in 1 mL of distilled water (a 100-fold dilution), depositing the solution onto a 200-mesh copper grid, and performing negative staining with 0.5% phosphotungstic acid (*w*/*v*; Sigma, St. Louis, MO, USA). Images were then acquired using a transmission electron microscope (JEM-2000EXII, JEOL Co., Tokyo, Japan).

### 2.8. Fourier Transform Infrared (FTIR) Spectroscopy Analysis

Fourier transform infrared spectroscopy (IRSpirit, Shimadzu, Japan) was utilized to examine bond formations and identify functional groups in HHPNP. The analyzed samples included hesperetin, HPBCD, PVP-K30, their physical mixture (1:15:12), as well as HHPNP in varying proportions. The samples were blended with anhydrous potassium bromide and scanned across a range of 400–4000 cm^−1^ for analysis.

### 2.9. X-Ray Diffraction (XRD) Analysis

The crystal structures of HSP, HHPNP, and the physical mixtures, were examined using an X-ray Diffractometer (Siemens D5000, Munich, Germany). The XRD analysis utilized Cu-Kα radiation as the source, with the equipment set to operate at 40 kV and 40 mA. Measurements were conducted at scanning angles (2*θ*) spanning from 5° to 50° at a rate of 1°/min.

### 2.10. In Vitro Antioxidant Activities of HHPNP

HSP was dissolved in ethanol and deionized water, respectively; while HHPNP used only deionized water. Each sample was then diluted for antioxidant testing. A 200 µM DPPH solution in ethanol with an optical density about 0.7 was prepared. In a 96-well plate, 100 µL of each sample was mixed with 100 µL DPPH solution, and kept in the dark at room temperature for 30 min. Absorbance was measured at 517 nm via microplate analyzer, and the results were expressed as free radical scavenging percentages and SC50 values. Equation (3) was employed to calculate the free radical scavenging effect.

The free radical scavenging effect (%) = [1 − (sample OD517nm/blank OD517 nm)] × 100%(3)

### 2.11. Dissolution Studies

The dissolution profiles were examined using the basket method detailed in USP XXIV, utilizing an eight-vessel dissolution tester (Model DT-8, New Taipei, Taiwan). The dissolution testing included four sample groups: 10 mg of HSP powder with HPBCD and PVPK30 physical mixture, HHPNP containing 10 mg of HSP equivalence, and encapsulated in gelatin capsules (Da-Ming Co., Taichung, Taiwan), and in HPMC capsules (Dah Feng Capsule Industry Co., Taichung, Taiwan), respectively. Six samples were used in each group for dissolution test. To mimic the physiological environment of the stomach (hydrochloric acid solution at pH 1.2) and small intestine (phosphate buffer at pH 6.8) as specified in USP XXIV, each sample was placed into 900 mL of the simulated solutions separately. The dissolution medium underwent continuous stirring at 50 rpm using a paddle apparatus to maintain even agitation. Aliquots were withdrawn at intervals of 5, 10, 15, 20, 25, 30, 45, 60, 90, and 120 min to track the dissolution behavior. Each 1 mL sample was passed through a 0.45 μm filter to remove particulates prior to analysis. The concentrations of HSP in the filtered aliquots were quantified using the established HPLC analytical method mentioned above.

### 2.12. Statistical Analysis

Data are expressed as mean ± standard deviation. One-way ANOVA, followed by post hoc LSD testing (SPSS 13, Chicago, IL, USA), was used to evaluate the means. Significance was set at *p* < 0.05.

## 3. Results and Discussion

### 3.1. Mechanism of Nanoparticle Formation of HHPNP

**Figure 2:**
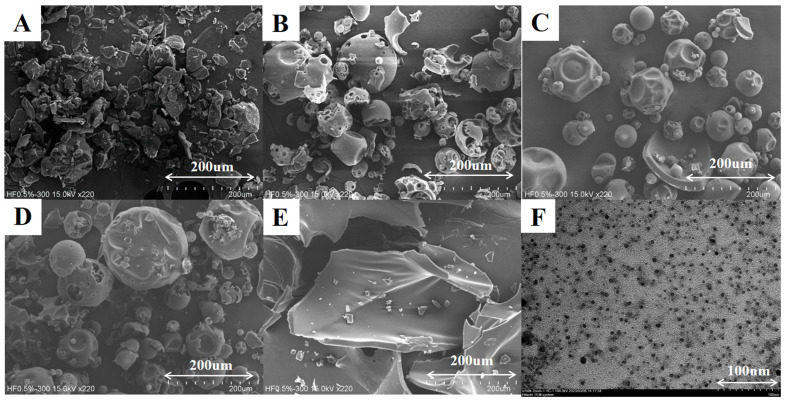
SEM images showing the surface morphology of (**A**) hesperetin (HSP), (**B**) HPBCD, (**C**) PVP-K30, (**D**) physical mixture (1:15:12), and (**E**) HHPNP (1:15:12); and (**F**) TEM image of HHPNP (1:15:12).

The successful incorporation of active nutraceutical ingredients (ANI) into delivery system dosage forms can be evaluated by examining various physicochemical properties. These assessments include surface microstructure analysis, which provides insights into the morphology, particle size, and surface characteristics of the delivery system. SEM is a well-established technique for directly examining the surface microstructure of ANI delivery systems, significantly contributing to the validation of molecular interactions, such as in hyperoside [13] and luteolin [14]. Figure 2 depicts the morphology of HSP, HPBCD, PVP-K30, physical mixture (1:15:12), and HHPNP (1:15:12) observed under a scanning electron microscope. It can be observed that HSP appears as irregular fragmented pieces with sizes ranging from 40 to 80 μm (Figure 2A). HPBCD exhibits hollow spherical structures (Figure 2B), while PVP-K30 appears as relatively smooth spheres with indentations (Figure 2C). The physical mixture (1:15:12) still shows the distinct features of HSP, HPBCD, and PVP-K30 (Figure 2D). In contrast, the HHPNP prepared through the co-precipitation method exhibits significantly different crystal morphology, appearing as large plate-like structures, deviating from the physical mixture (1:15:12) (Figure 2E).

As shown in Figure 2F, TEM imaging of HHPNP (1:15:12) reveals spherical nanoparticles when dispersed in water. This morphology aligns with the hydrodynamic size measured by the Zetasizer Nano-ZS ZEN3600, with an average particle size of 14.87 ± 0.49 nm (Table 2). In addition, the particles exhibit uniform dispersion, indicating stability. Table 2 shows that the preparation of the HSP into a nanoparticle delivery system effectively reduces the average particle size, thereby enhancing the surface area. However, the average particle size and polydispersity index did not vary significantly with the increase in PVPK30 concentration. This may be attributed to the fact that PVPK30 provides sufficient stability and uniformity to the formulation at a certain proportion. Similar results have been reported in other studies as well [15].

### 3.2. Encapsulation Mechanism of Hesperetin in Nanoparticle Delivery Systems

**Figure 3:**
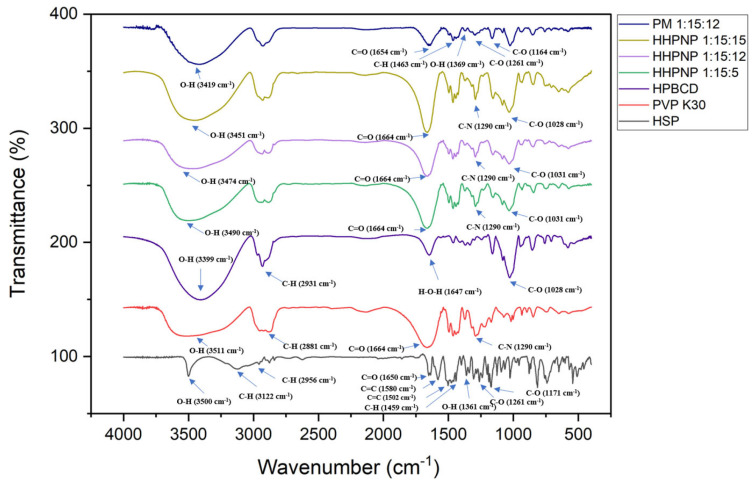
FTIR spectra of HSP, HPBCD, PVP-K30, physical mixture (PM 1:15:12), and HHPNP inclusion complexes with different ratios (1:15:5, 1:15:12, and 1:15:15).

The FTIR spectra in Figure 3. show the characteristic functional group absorption bands of HSP, such as the phenolic O-H stretching band (3500 cm^−1^), aromatic ring C-H stretching band (3122 cm^−1^), alkyl C-H stretching band (2956 cm^−1^), C=O stretching band (1650 cm^−1^), aromatic hydrocarbon C=C stretching band (1500~1600 cm^−1^), alkyl C-H bending band (1459 cm^−1^), phenolic O-H bending band (1361 cm^−1^), alkyl aryl ether C-O stretching band (1261 cm^−1^), and aliphatic ether C-O stretching band (1171 cm^−1^). In addition, HPBCD exhibits O-H stretching band (3399 cm^−1^), methyl C-H stretching band (2931 cm^−1^), H-O-H stretching band (1647 cm^−1^), and aliphatic ether C-O stretching band (1028 cm^−1^) and PVPK30 shows alkyl C-H stretching band (2881 cm^−1^), C=O stretching band (1664 cm^−1^), and C-N stretching band (1290 cm^−1^). PVPK30 does not have an OH group, but it exhibits a broad O-H stretching band (3511 cm^−1^), indicating its hygroscopic nature and the presence of moisture. This phenomenon has also been observed in resveratrol [16] and myricetin [17] formulations.

The FTIR results suggest that after the formation of the nanoparticle delivery system, the O-H absorption band of HPBCD (3399 cm^−1^) in the HHPNP shifts to a higher frequency (3451 cm^−1^) and becomes broader, indicating the molecular hydrogen bonding between HSP and HPBCD. The characteristic absorption bands of HSP are also partially masked, such as alkyl aryl ether C-O stretching band (1261 cm^−1^), aliphatic ether C-O stretching band (1171 cm^−1^), C-H bending band (1459 cm^−1^), and carbonyl stretching band C=O (1650 cm^−1^). The peaks of all three HHPNP are covered by the γ-lactam C=O stretching band (1664 cm^−1^) and C-N stretching band (1290 cm^−1^) of PVPK30, as well as the aliphatic ether C-O stretching band (1028 cm^−1^) of HPBCD. In the physical mixture group, the O-H band of HPBCD does not show a significant shift to a higher frequency, and the characteristic absorption peaks of HSP are not significantly masked, such as the C=O stretching band (1654 cm^−1^), C-H bending band (1463 cm^−1^), phenolic O-H rocking band (1369 cm^−1^), alkyl aryl ether C-O stretching band (1261 cm^−1^), and aliphatic ether C-O stretching band (1164 cm^−1^). These observations indicate that the physical mixture without nano-preparation clearly does not encapsulate HSP, as its characteristic peaks are not masked and there is no formation of intermolecular hydrogen bonds. After the preparation using the co-precipitation method, HSP is successfully encapsulated by HPBCD, forming the nanoparticle delivery system, which masks the characteristic absorption bands of HSP.

### 3.3. Effect of Particle Size Reduction and Amorphous Transformation on HSP Water Solubility

Table 3 highlights that all formulation ratios yield over 80%, signifying negligible material loss during the process. There is dramatic improvement in HSP’s water solubility upon incorporation into the formulation, and among the tested ratios, the 1:15:12 formulation achieves the highest water solubility. Additionally, the solubility at the 1:15:15 ratio ranks lower than 1:15:12, reinforcing findings from prior studies suggesting optimal drug solubility is attained at a specific polymer concentration, while exceeding this threshold can actually diminish solubility [18]. These findings confirm that HSP has been effectively encapsulated within HPBCD and PVPK30, creating a nanoparticulate delivery system. This advancement not only expands the compound’s surface area but significantly boosts its water solubility. Taking into account both the remarkable enhancement in solubility and the cost-effectiveness, the study identifies the 1:15:12 formulation as the ideal choice for further development and research.

**Figure 4:**
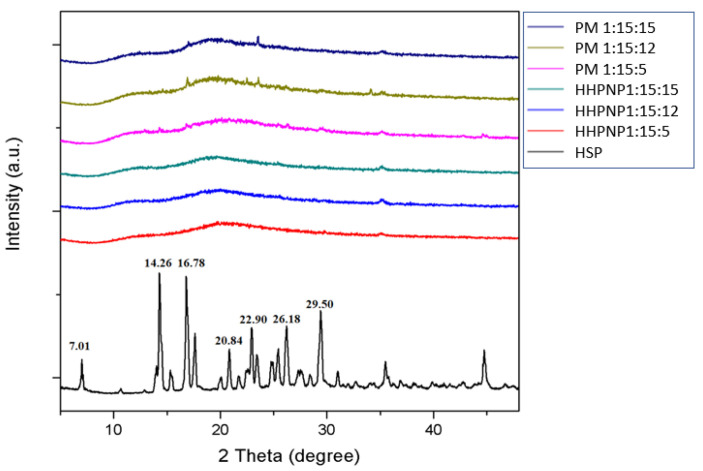
XRD patterns of hesperetin (HSP), physical mixtures (PM, 1:15:5, 1:15:12, and 1:15:15), and HHPNP inclusion complexes with different ratios (1:15:5, 1:15:12, and 1:15:15).

Figure 4 further highlighted the XRD pattern of HSP, which exhibited multiple sharp diffraction peaks at 2*θ* angles of 7.01°, 14.26°, 16.78°, 20.84°, 22.90°, 26.18°, and 29.50°. These distinct peaks clearly indicated the crystalline nature of HSP. Conversely, the excipients HPBCD and PVPK30 displayed no diffraction peaks, signifying their amorphous nature. This contrast in structural characteristics allowed the process of HSP encapsulation to be effectively analyzed via XRD, leveraging the amorphous properties of HPBCD and PVPK30 [19]. In the physical mixture samples at different ratios, diffraction peaks corresponding to HSP, specifically at 14.26°, 16.78°, 20.84°, 22.90°, 26.18°, and 29.50°, were still detected. This observation confirmed that encapsulation by the excipients was incomplete. On the other hand, as revealed by the results, HHPNP samples across various ratios displayed a complete absence of these HSP peaks. This absence demonstrated the successful encapsulation of HSP by the excipients, effectively converting it from a crystalline form into an amorphous state. Consequently, this structural transformation resulted in greatly enhanced water solubility. These findings not only validate the effectiveness of the encapsulation method but also affirm the successful formulation of the nanoparticle delivery system.

### 3.4. Effect of Nanoparticle Formulation on Antioxidant Activity

**Figure 5:**
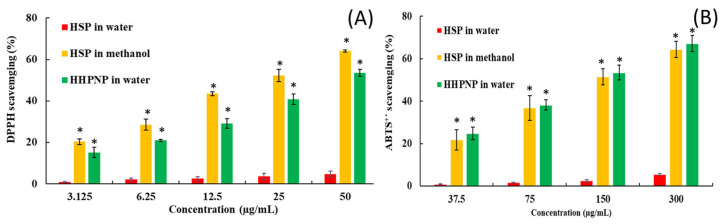
The antioxidant activity of HSP and HHPNP. (**A**) DPPH scavenging effect, and (**B**) ABTS^+^ scavenging effect. * *p* < 0.05 as compared with the HSP in water group.

This study investigated the antioxidant properties of HSP using the DPPH and ABTS^+^ radical scavenging assays across three distinct formulations: HSP in water, HSP-encapsulated nanoparticles (HHPPN) in water, and HSP dissolved in methanol. The findings revealed that HSP in water exhibited minimal antioxidant activity in both assays, achieving scavenging rates of less than 10% even at the highest concentrations tested. Moreover, its SC_50_ could not be determined (Figure 5). This insufficient activity was linked to HSP’s exceedingly poor solubility in aqueous environments, which severely restricts its capacity to counteract free radicals. Conversely, when dissolved in methanol, HSP demonstrated markedly improved antioxidant activity. In the DPPH assay, it achieved around 40% scavenging at a concentration of 12.5 µg/mL and exceeded 70% activity at 50 µg/mL, with an SC_50_ of 15.00 ± 0.82 µg/mL. A lower SC_50_ value indicates more potent antioxidant activity. Similarly, in the ABTS^+^ assay, HSP in methanol displayed strong dose-dependent antioxidant efficacy, with scavenging activity reaching nearly 70% at 300 µg/mL and an SC_50_ of 137.45 ± 16.48 µg/mL. These results clearly highlight the pivotal influence of the solvent on enhancing HSP’s bioactive potential. The HHPPN formulation, which utilizes nanoparticle-assisted encapsulation, showed a notable improvement in antioxidant activity compared to HSP in water. HHPPN achieved approximately 60% DPPH scavenging at 50 µg/mL and recorded an SC_50_ value of 27.67 ± 6.02 µg/mL. In the ABTS^+^ assay, it delivered comparable results to methanolic HSP, reaching around 70% scavenging at 300 µg/mL, with an SC_50_ of 131.74 ± 12.46 µg/mL. These findings reveal that nanoparticle encapsulation can effectively enhance the antioxidant activity of HSP in water, making it a viable alternative to organic solvents for delivering bioactive compounds in pharmaceutical and nutraceutical applications.

### 3.5. Dissolution Profiles of HSP Nanoparticle Powders in Gelatin and HPMC Capsules

**Figure 6:**
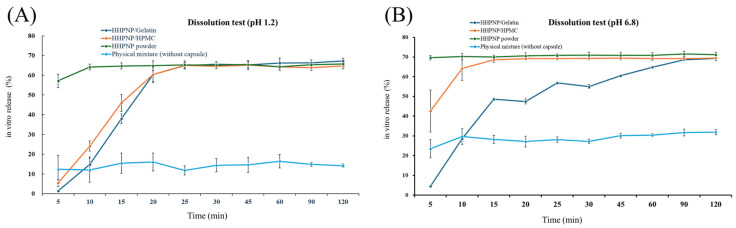
The in vitro dissolution profiles of HHPNP, HSP physical mixture (HSPPM), HHPNP filled into non-vegetarian capsule (HHPNP/Gelatin) and vegetarian capsule (HHPNP/HPMC) in (**A**) simulated gastric conditions (pH 1.2) and (**B**) simulated intestinal conditions (pH 6.8).

Nowadays, most nutraceuticals on the health food market use powder or capsule forms as sources of nutritional supplementation. Since HSP is classified as a BCS Class II compound, its absorption is primarily limited by its poor solubility and dissolution profile. In the present study, an in vitro dissolution release test was conducted in a simulated human gastric acid (pH 1.2) and intestinal fluid (pH 6.8) environment to investigate the release rate and dissolution profile of HSP nanoparticle formulation (HHPNP), HSP physical mixture (HSPPM), HHPNP filled into non-vegetarian capsule (gelatin composition) and vegetarian capsule (HPMC composition), respectively. As shown in Figure 6A, the release rate of HSPPM in pH 1.2 is extremely low, reaching only about 10% of the release amount in 120 min, and the overall release curve is gentle, indicating that its solubility and release capacity are limited in acidic environments, making it difficult to effectively release active ingredients. As indicated by the Henderson-Hasselbalch equation, HSP (pKa ≈ 7.5) predominantly remains in its undissociated molecular state within the simulated gastric environment (pH1.2). Jin et al. mentioned that the undissociated form of HSP possesses extremely low water solubility (1.35 µg/mL) [20], this accounts for the HSPPM achieving only approximately a 10% release rate at pH 1.2. In contrast, HHPNP successfully addressed the solubility challenges of HSP in acidic conditions by utilizing the encapsulation properties of HPBCD and the solubilizing effect of PVPK30. It exhibited a markedly improved dissolution release profile at pH 1.2 and pH 6.8, achieving a rapid release rate within the first 5 min and surpassing 60% release in approximately 10 min. This demonstrates the potential of nanotechnology to significantly enhance the swift and efficient release of HSP in acidic environments. HHPNP containing HPBCD can establish a stable host-guest inclusion complex with HSP by encapsulating the hydrophobic HSP molecules within the hydrophobic cavity of the HPBCD. Simultaneously, the hydrophilic outer hydroxyl groups interact with water molecules, resulting in a soluble complex. This inclusion mechanism notably improves the solubility of the drug without altering its chemical structure and enhances its in vitro release rate [21]. Liu et al. also mentioned that cyclodextrin inclusion complexes exhibit excellent hydrophilicity and rapid solubility, significantly enhancing the solubilization effect and the application value of active ingredients in food and pharmaceuticals [13]. In food, HPBCD inclusion complexes not only prevent the oxidative degradation of active ingredients and maintain their bioactivity but also extend the shelf life of food. More importantly, they enable controlled release and targeting characteristics, enhance the bioavailability of active ingredients, and reduce side effects. These findings provide a theoretical foundation for the design of functional foods containing active ingredients and the development of innovative nutraceuticals delivery systems. Moreover, PVPK30 is an amphiphilic polymer with solubilizing and stabilizing properties, often used to improve the solubility of poorly soluble nutraceutical components such as curcumin [22] and resveratrol [23]. HHPNP utilizes PVPK30 to enable hydrogen bond interactions with the molecular structure of HSP, successfully forming an amorphous solid dispersion. This process effectively inhibits the crystallization of HSP while preventing nanoparticle aggregation through spatial hindrance effects. More importantly, the particle size of HHPNP is 14.87 ± 0.49 nm, significantly smaller than the particle size of HSP powder (>1 μm), thus enhancing the specific surface area of HSP and improving its water solubility and in vitro dissolution profile. Taken together, HHPNP significantly enhanced the release rate of HSP, increasing the release from only 10% within 120 min in the physical mixture form to over 60% within 10 min in the nanoparticle formulation. This nanotechnology plays a crucial role in improving the in vitro release of HSP.

Additionally, in vitro dissolution tests conducted on HHPNP filled in gelatin capsules (non-vegetarian) and HPMC capsules (vegetarian) in simulated gastric environment revealed that roughly 65% of the HHPNP was released within the initial 25 min, followed by a stable release phase. Initially, both capsule types exhibited a lag period due to shell disintegration. Once disintegrated, HHPNP was rapidly released, aligning with the release profile of the non-encapsulated form. In simulated intestinal fluid, it can be observed that HPMC capsule hydrates and swells rapidly, showing a faster release as compared to the gelatin capsule, as it forms a denser, more cohesive gel network at neutral to slightly basic pH, slowing down water penetration and shell disintegration relative to that of HPMC at pH 6.8. Comparatively, the T50% values (time required to achieve 50% release) for gelatin and HPMC capsules at pH 1.2 were 17.8 and 16.8 min, respectively, with no significant difference (*p* > 0.05). While at pH 6.8, the T50% values for gelatin and HPMC capsules were 17.6 and 7.5 min, respectively, corroborating the rapid hydration of HPMC in this medium. Both capsules rapidly disintegrated in the simulated environment, matching the HHPNP in release at 120 min (65% at pH 1.2 and 70% at pH 6.8). These comparable profiles indicate the formulation’s stability and adaptability across capsule variants, leading to comparable HHPNP release kinetics.

## 4. Conclusions

To summarize, this study reveals that the HSP nanoparticle formulation significantly enhances the in vitro release rate of HSP, with the potential to improve its oral bioavailability. Notably, the total release of HHPNP remains largely unaffected whether filled in gelatin or HPMC capsules, confirming that both capsule types are suitable for developing nanoparticle oral dosage forms. This versatility makes them ideal for accommodating both vegetarian and non-vegetarian preferences. Furthermore, the findings highlight that HHPNP formulations can be packaged directly in aluminum foil as a food-grade dosage form for quicker absorption, offering valuable insights for the food industry to consider HSP for future oral nutraceutical applications.

## Figures and Tables

**Table 1:** Composition ratios of hesperetin (HSP), HPBCD, and PVPK30 used for the preparation of hesperetin-loaded nanoparticles (HHPNP).

Ratio	HSP (mg)	HPBCD (mg)	PVP-K30 (mg)
1:15:5	20	300	100
1:15:12	20	300	240
1:15:15	20	300	300

**Table 2:** Particle size and polydispersity index of HSP and HHPNP formulations with different ratios.

HSP: HPBCD:PVPK30	Particle Size (nm)	Polydispersity Index
Hesperetin	>1000	-
1:15:5	13.51 ± 0.26 *	0.255 ± 0.01
1:15:12	14.87 ± 0.49 *	0.258 ± 0.01
1:15:15	14.03 ± 0.22 *	0.226 ± 0.01

Data are expressed as the mean ± standard deviation (SD), and all the data were collected in triplicate. * *p* < 0.05 compared with HSP.

**Table 3:** Water solubility, encapsulation efficiency, and yield of HHPNP prepared with different ratios of HSP:HPBCD:PVP-K30.

HSP: HPBCD:PVPK30	Water Solubility(μg/mL)	Encapsulation Efficiency (%)	Yield (%)
Hesperetin	0.96 ± 0.12 *	-	-
1:15:5	426.05 ± 19.70 *	81.42 ± 1.41	85.23 ± 5.0
1:15:12	794.00 ± 25.73 *	90.41 ± 1.32	88.55 ± 4.19
1:15:15	682.95 ± 8.379 *	90.73 ± 1.32	83.78 ± 5.24

Data are expressed as the mean ± standard deviation (SD), and all the data were collected in triplicate. * *p* < 0.05 compared with HSP.

## Data Availability

All data presented in the study are available upon request from the corresponding author (flyen@kmu.edu.tw).
